# Vitamin D deficiency in pregnancy and the risk of preterm birth: a nested case–control study

**DOI:** 10.1186/s12884-023-05636-z

**Published:** 2023-05-06

**Authors:** Tashnia Tahsin, Rasheda Khanam, Nabidul Haque Chowdhury, A. S. M. Tarik Hasan, Md. Biplob Hosen, Sayedur Rahman, Anjan Kumar Roy, Salahuddin Ahmed, Rubhana Raqib, Abdullah H. Baqui

**Affiliations:** 1 Department of Medicine, West Virginia University, Morgantown, WV USA; 2 Department of International Health, Johns Hopkins Bloomberg School for Public Health, Baltimore, MD USA; 3 Projahnmo Research Foundation, Banani, Dhaka-1213 Bangladesh; 4 International Center for Diarrhoeal Disease Research, Dhaka, Bangladesh; 5 Department of Women’s and Children’s Health, Uppsala University, Uppsala, Sweden

**Keywords:** Preterm birth, Serum vitamin D, Nested case–control study, Bangladesh

## Abstract

**Background:**

Each year, an estimated 15 million babies are born preterm. Micronutrient deficiencies, including vitamin D deficiency (VDD), are common in many low- and middle-income countries (LMICs), and these conditions are often associated with adverse pregnancy outcomes. Bangladesh experiences a high prevalence of VDD. The country also has a high preterm birth (PTB) rate. Using data from a population-based pregnancy cohort, we estimated the burden of VDD during pregnancy and its association with PTB.

**Methods:**

Pregnant women (*N* = 3,000) were enrolled after ultrasound confirmation of gestational age at 8–19 weeks of gestation. Trained health workers prospectively collected phenotypic and epidemiological data at scheduled home visits. Trained phlebotomists collected maternal blood samples at enrollment and 24 -28 weeks of gestation. Aliquots of serum were stored at -80^0^ C. We conducted a nested case–control study with all PTB (*n* = 262) and a random sample of term births (*n* = 668). The outcome, PTB, was defined as live births < 37 weeks of gestation, based on ultrasound. The main exposure was vitamin D concentrations of 24–28 weeks maternal blood samples. The analysis was adjusted for other PTB risk factors. Women were categorized as VDD (lowest quartile of 25(OH)D; <  = 30.25 nmol/L) or not deficient (upper-three quartiles of 25(OH)D; > 30.25 nmol/L). We used logistic regression to determine the association of VDD with PTB, adjusting for potential confounders.

**Results:**

The median and interquartile range of serum 25(OH)D was 38.0 nmol/L; 30.18 to 48.52 (nmol/L). After adjusting for co-variates, VDD was significantly associated with PTB [adjusted odds ratio (aOR) = 1.53, 95% confidence interval (CI) = 1.10 – 2.12]. The risk of PTB was also higher among women who were shorter (aOR = 1.81, 95% CI: 1.27–2.57), primiparous (aOR = 1.55, 95% CI = 1.12 – 2.12), passive smokers (aOR = 1.60, 95% CI = 1.09 – 2.34), and those who received iron supplementation during pregnancy (aOR = 1.66, 95% CI: 1.17, 2.37).

**Conclusion:**

VDD is common in Bangladeshi pregnant women and is associated with an increased risk of PTB.

## Background

Globally, an estimated 15 million babies are born preterm each year, which is increasing. Preterm birth (born before 37 completed weeks of gestation) and its complications are the leading cause of neonatal deaths [1, 2] and deaths among children under the age of 5 years, accounting for approximately one million child deaths each year [3, 4]. Many surviving preterm babies experience a lifetime disability, including learning disabilities and visual and hearing problems [5]. Low- and middle-income countries (LMICs), such as those in Southeast Asia and Sub-Saharan Africa, have a significantly higher burden of preterm birth (PTB) [6] National-level data on PTB in Bangladesh is unavailable. Based on modeling, Chawanpaiboon S. et al., estimated that the preterm birth rate in Bangladesh is 19.1% [7].

**Fig. 1:**
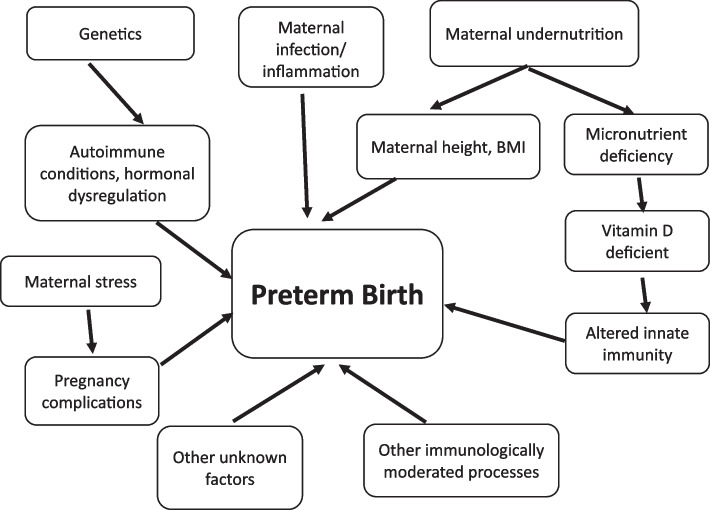
Conceptual framework: factors associated with preterm birth and the possible pathways

The ability to find markers that correlate with PTB can aid in decreasing child morbidity and mortality rates. PTB is of multifactorial origin, partially associated with immunologic, genetic, nutritional, and environmental factors [8–10]. The other factors include infection or inflammation, pregnancy complications, including uteroplacental ischemia or hemorrhage, previous preterm birth, periodontal disease, uterine overdistension, stress, and other immunologically mediated processes [11, 12]. Figure 1 shows the factors associated with preterm birth and the possible pathways. The attribution of these factors has not yet been well elucidated.

Micronutrient deficiencies, including vitamin D deficiency (VDD), are common in many LMICs, and these deficiencies are often associated with adverse pregnancy outcomes [13, 14]. Pregnant women have a higher demand for micronutrients, and complications can arise from maternal micronutrient deficiency [15]. Vitamin D is an important micronutrient that regulates calcium and phosphate metabolism; it is essential for building bones. It is also a necessary part of the innate immune system as it aids in producing antimicrobial peptides [16]. Inadequate dietary intake, conditions that prevent the skin from producing enough vitamin D, and other factors that interfere with vitamin D metabolism or absorption can all contribute to VDD [17]. The current literature examining the association of VDD and PTB is conflicting. Some studies observed that low maternal vitamin D concentration was correlated with adverse outcomes in pregnancy, including PTB, low birth weight, gestational diabetes, and pre-eclampsia [18–23]. However, other studies did not show such association [24, 25].

VDD is highly prevalent in Bangladesh, with 66% to 94.2% adult women in their childbearing age experiencing the deficiency [26]. Bangladesh also experiences one of the highest PTB rates [7]. According to a systematic review published in Lancet Global Health, Bangladesh is one of the top five countries that experience the largest number of PTB [7]. India, China, Nigeria, Bangladesh, and Indonesia accounted for 57.9 million (41.4%) of 139·9 million live births and 6.6 million (44.6%) of 14.8 million PTB globally in 2014 [7, 27, 28]. The high burden of VDD as well as PTB in Bangladesh, makes the country a suitable place to further investigate if a correlation exists between the two. In this study, we aimed to investigate the relationship between Serum 25(OH)D concentrations and PTB among pregnant women in a rural area of Bangladesh.

## Methods

### Study design, setting, and data

This is a nested case–control study that uses data from a population-based cohort of pregnant women and their children; the study is known as the Alliance for Maternal and Newborn Health Improvement (AMANHI). As part of AMANHI, we established a biorepository and enrolled 3,000 pregnant women between 2014 and 2018 in two sub-districts of Sylhet district in northeast Bangladesh and followed them until day 42–60 postpartum. The details of the study methodology were published earlier [29]. Trained community health workers (CHWs) with a minimum of ten-grade education collected data after obtaining written informed consent. Pregnancies were identified through 2-monthly home visits and confirmed by strip–based pregnancy tests administered by CHWs and dated through ultrasound scans carried out by trained ultrasonologists between 8 and 19 weeks of gestation. CHWs made three antenatal home visits (at 8–19 weeks, 24–28 weeks, and 32–36 weeks of gestation) and two postnatal home visits (< 7 days and at 42–60 days). During these visits, the CHWs collected detailed phenotypic, socio-demographic, and epidemiological data from the pregnant women.

### Sample collection

Maternal blood samples were collected twice during pregnancy (8–19 weeks and 24–28 weeks or 32–36 weeks of gestation) and once during the postpartum period (42–60 days postpartum). The second pregnancy sample was collected from about three-fourths of the randomly selected women. The third sample was collected from women who were not selected for the second blood sample. The blood samples were collected by trained phlebotomists at the study clinic, and serum samples were separated by centrifugation, aliquoted, and stored at -80 ℃ using standard procedures [30].

### Population, cases and controls

**Fig. 2:**
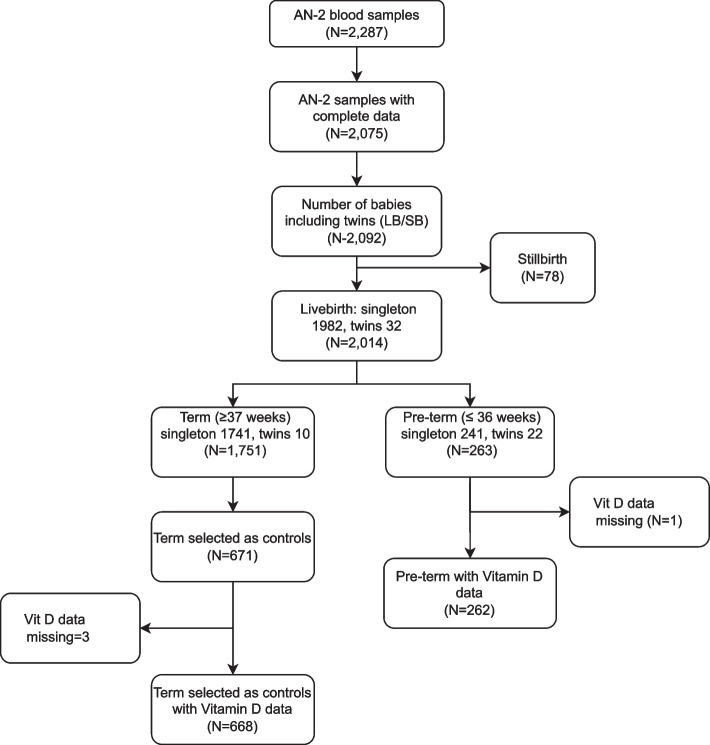
Study flow chart

For this study, we considered all women who had the second antenatal blood drawn (*n* = 2,287), had at least one antenatal and one postnatal visit and the pregnancy outcome data (*n* = 2,075), and had a live-born baby (*n* = 2,014). In this nested case–control study, all women who had a PTB (between 24 and < 37 weeks of gestation) were included as cases (*n* = 263) and a random sample of term births were included as controls (*n* = 671). Vitamin D was measured from cases and controls only. Vitamin D measurement was missing in one case and three control mothers; these mothers were excluded from the analysis. Thus, 262 cases and 668 controls were included in the analysis (Fig. 2).

### Vitamin D assay

The frozen serum samples were transferred to the Immunobiology, Nutrition and Toxicology laboratory of the international center for diarrhoeal disease research Bangladesh (icddr,b) for analysis. Serum 25(OH)D concentrations were measured by electrochemiluminescence immunoassay (ECLIA) with Roche automated immunoassay analyzers Cobas e601 using a commercial kit (Roche Diagnostics, GmbH, 68305 Mannheim, Germany) according to the manufacturer’s instruction. The method for vitamin D was standardized against LC MS/MS which in turn was standardized to the NIST standard. The measuring range for 25(OH)D was 7.50‑175 nmol/L. Commercial control based on human serum in two concentration ranges was run in each lot/day to monitor the accuracy and precision of this assay.

### Measurements

The outcome, PTB, was defined as live births < 37 weeks of gestation, based on ultrasound. The main exposure was vitamin D concentrations of 24–28 weeks maternal blood samples. Using Serum 25(OH)D concentrations, we categorized women into quartiles [lowest quartile (≤ 30.25 nmol/L), 3rd quartile (30.25–37.99 nmol/L), 2nd quartile (38.0–48.52 nmol/L), and highest quartile (≥ 48.53 nmol/L)]. Table 1 showed that about 75% of women had an insufficient vitamin D level (< 48.53 nmol). We then examined the association of vitamin D concentrations with the primary outcome, PTB and observed that compared to the highest quartile of vitamin D, all other quartiles had a higher risk of PTB, but the risks were only marginally higher in the second and 3rd quartiles. The lowest quartile had a 67% significantly higher risk of PTB. We then combined the upper three quartiles of vitamin D as ‘not deficient’ [25(OH)D ≥ 30.25 to ≥ 48.53 nmol/L] and the lowest quartile as vitamin D ‘deficient’ [25(OH)D ≤ 30.25 nmol/L]. We used these two categories in all subsequent analysis (Table 1).

**Table 1:** Association of preterm birth and vitamin D concentrations

	**Total** ***N* = 930 (%)**	**Term** ***n* = 668 (%)**	**Preterm** ***n* = 262 (%)**	**OR (95% CI)**	***P*-value**
**25(OH)D concentration (quartiles)**					
Highest quartile (≥ 48.53 nmol/L)	232 (25.0)	177 (26.5)	56 (21.4)	Reference	
2^nd^ quartile (38.0–48.52 nmol/L)	233 (25.0)	172 (25.8)	60 (22.9)	1.13 (0.74–1.72)	0.574
3^rd^ quartile (30.25–37.99 nmol/L)	232 (25.0)	167 (25.0)	66 (25.2)	1.24 (0.82–1.88)	0.306
Lowest quartile (≤ 30.25 nmol/L)	233 (25.0)	152 (22.8)	80 (30.5)	1.67 (1.12–2.51)	0.013
**25(OH)D concentrations (two categories)**					
Not deficient (upper three quartiles)	697 (75%)	516 (77.2)	182 (69.5)	Reference	
Deficient (lowest quartile)	233 (25%)	152 (22.8)	80 (30.5)	1.49 (1.08–2.05)	0.014

Maternal age was categorized into < 30 and ≥ 30 years. Parity was categorized as 0/primiparous, 1–3, and ≥ 4 children. Maternal and paternal education was categorized into 0–5 years and > 5 years of schooling. Maternal height was categorized into height < 145 cm and >  = 145 cm. Household crowding index was created by dividing the number of persons by number of sleeping rooms. We then categorized the household crowding into <  = 2 and > 2. Using principal component analysis, we calculated scores for household wealth based on housing materials and household contents. The households were divided into tertiles using the wealth scores.

### Sample size and statistical analysis

Since the number of cases were fixed, using Kelsey et al. [31] formula for sample size calculation for unmatched case–control study, we calculated the number of controls required per case to detect an Odd Ratio of 1.5 with the following parameters: proportion of controls with exposure = 20%; proportion of cases with exposure = 30%; two-sided confidence interval = 95%, and power = 80%. The number of controls required per case was 2.5. Accordingly, we selected 671 controls for 262 cases.

We conducted two sets of bi-variate analyses using Pearson’s chi-squared test for independence. First, we examined the association of vitamin D concentrations (deficient vs not deficient) with the mother’s age, education, occupation, maternal height, tobacco consumption, husband’s education, husband’s occupation, household crowding, and household wealth index. In the second set of bivariate analysis, we examined the association of infant’s preterm or term status with vitamin D concentrations and selected socioeconomic, demographic, and care-seeking characteristics of mothers. Multivariable logistic regression was used to calculate unadjusted and adjusted odds ratios (aORs) and 95% confidence intervals (CIs) to identify factors significantly associated with PTB. Variables with a p-value of < 0.2 in the bivariate analyses with PTB were included in the multivariable logistic regression model. We used Stata V.17 to analyze the data (StataCorp 2017).

## Results

The association of maternal serum vitamin D concentrations with selected characteristics of mothers and households is presented in Table 2. The mother’s age, education, and household crowding were significantly associated with vitamin D concentrations. Younger mothers, age < 30 years and those with education > 5 years were more likely to have vitamin D deficiency (Table 2).

**Table 2:** Distribution of maternal serum vitamin D concentrations by selected characteristics of mothers and households

**Characteristics**		**Vitamin D concentrations**		***P*-value**
	**Total**	**Not deficient**	**Deficient**	
	***N* = 930**	***n* = 698 (%)**	***n* = 232 (%)**	
**Mother’s age in years**				
< 30	816	602 (86.2)	214 (92.2)	0.016
≥ 30	114	96 (13.8)	18 (7.8)	
**Mother’s education**				
0–5 years	433	353 (50.6)	80 (34.5)	0.001
> 5 years	497	345 (49.4)	152 (65.5)	
**Mother’s occupation**				
Housewife	910	684 (98.0)	226 (97.4)	0.597
Working/employed	20	14 (2.0)	6 (2.6)	
**Mother’s BMI**				
**Mother’s height**				
**< 145 cm**	182	145 (20.8)	37 (15.9)	0.109
**≥ 145 cm**	748	553 (79.2)	195 (84.1)	
**Tobacco consumption during pregnancy**				
No (Never, Quit pre/during pregnancy)	769	580 (83.1)	189 (81.5)	0.57
Yes (currently sniffing/chewing)	161	118 (16.9)	43 (18.5)	
**Husband’s education**				
0–5 years	652	499 (71.5)	153 (65.9)	0.110
> 5 years	278	199 (28.5)	79 (34.1)	
**Husband's occupation**				
Govt/private/self-employed (possibly in-door)	260	193 (27.7)	67 (28.9)	0.718
Daily wage/farming/other/does not work (possibly out-door)	670	505 (72.3)	165 (71.1)	
**Household crowding**				
**≤ 2**	663	485 (69.5)	178 (76.7)	0.035
**> 2**	276	213 (30.5)	54 (23.3)	
**HH wealth index**				
Poorest	310	233 (33.4)	77 (33.2)	0.932
Middle	312	232 (33.2)	80 (34.5)	
Richest	308	233 (33.4)	75 (32.3)	

In bivariate analysis, the mother’s serum vitamin D concentrations, mother’s height, parity, iron supplementation during pregnancy, and passive smoking were associated with the infant’s term and preterm birth status (Table 3).

**Table 3:** Distribution of infant’s preterm and term status by selected socioeconomic, demographic, and care-seeking characteristics of mothers

**Variables**	**Total**	**PTB (< 37 Weeks)**	**Term (≥ 37 weeks)**	***P*-value**
	***N* = 930**	***n* = 262 (%)**	***n* = 668 (%)**	
**Vitamin-D concentrations**				
Not deficient (upper three quartiles)	698	182 (69.5)	516 (77.2)	0.014
Deficient (lowest quartiles)	232	80 (30.5)	152 (22.8)	
**Sex of the baby**				
Boy	458	132 (50.4)	326 (48.8)	0.665
Girl	472	130 (49.6)	342 (51.2)	
**Mother’s age in years**				
< 30	816	233 (88.9)	583 (87.3)	0.489
≥ 30	114	29 (11.1)	85 (12.7)	
**Mother’s education**				
0–5 years	433	118 (45.0)	315 (47.2)	0.560
> 5 years	497	144 (55.0)	353 (52.8)	
**Mother’s height**				
**< 145 cm**	182	70 (26.7)	112 (16.8)	0.001
**> = 145 cm**	748	192 (73.3)	556 (83.2)	
**Parity**				
0/Primi	302	101 (38.5)	201 (30.1)	0.023
1–3	519	128 (48.9)	391 (58.5)	
≥ 4	109	33 (12.6)	76 (11.4)	
**Place of delivery**				
Home	238	72 (27.5)	166 (24.9)	0.408
Facility	692	190 (72.5)	502 (75.1)	
**Skilled delivery assistance**				
Skilled birth attendant	911	256 (97.7)	655 (98.1)	0.739
Untrained	19	6 (2.3)	13 (1.9)	
**Taking iron tablets during pregnancy**				
No	250	52 (19.8)	198 (29.6)	0.002
Yes	680	210 (80.2)	470 (70.4)	
**Tobacco consumption during pregnancy**				
No (Never, Quit pre/during pregnancy)	769	210 (80.2)	559 (83.7)	0.201
Yes (currently sniffing/chewing)	161	52 (19.8)	109 (16.3)	
**Passive or indirect smoking during pregnancy**				
No	197	44 (16.8)	153 (22.9)	0.04
Yes	733	218 (83.2)	515 (77.1)	
**Chewing betel leaf during pregnancy**				
No	543	155 (59.2)	388 (58.1)	0.764
Yes	387	107 (40.8)	280 (41.9)	
**Religion**				
Muslim	808	230 (87.8)	578 (86.5)	0.609
Others	122	32 (12.2)	90 (13.5)	
**Husband’s education**				
0–5 years	652	188 (71.8)	464 (69.5)	0.492
> 5 years	278	74 (28.2)	204 (30.5)	
**Household crowding**				
**< = 2**	663	188 (71.8)	475 (71.1)	0.844
**> 2**	267	74 (28.2)	193 (28.9)	
**HH wealth index**				
Poorest	310	85 (32.4)	225 (33.7)	0.924
Middle	312	90 (34.4)	222 (33.2)	
Richest	308	87 (33.2)	221 (33.1)	

In the unadjusted logistic regression analysis examining the factors associated with PTB, mothers with VDD (OR, 95% CI: 1.49, 1.08–2.05), mother’s height (OR, 95% CI: 1.81, (1.29–2.54), primiparity (OR, 95% CI: 1.53, 1.12–2.10), mothers with history of passive smoking during pregnancy (OR, 95% CI: 1.47, 1.02–2.13), and mothers who received iron supplements (OR, 95%CI: 1.70 (1.20–2.40) showed a higher risk of PTB (Table 4). The risk of PTB associated with VDD remained similar after adjusting for other covariates that were significant in the bivariate analysis. Maternal serum VDD was associated with about 1.5 times higher risk of PTB (aOR, 95% CI: 1.53, 1.10 to 2.12). The risk of PTB was about 2 times higher among mothers with height < 145 cm (aOR, 95% CI: 1.81, 1.27–2.57) compared mothers with height >  = 145 cm (Table 4). Compared to mothers with 1–3 children, primiparous mothers had about 1.5 times higher risk of PTB (aOR, 95% CI: 1.55, 1.12 -2.12) (Table 4). Mothers who consumed iron during pregnancy showed a 66% higher risk of PTB (aOR, 95% CI: 1.66, 1.17- 2.37) compared to mothers who did not (Table 4). In comparison to mothers who did not report passive smoking, those who did report it had 1.60 times (aOR, 95% CI: 1.60, 1.09–2.34) increased risk of PTB (Table 4).

**Table 4:** Risk of preterm births in vitamin D deficient mothers after adjusting for co-variates

**Characteristics**	**Preterm birth**	
	**Unadjusted OR** **95%CI**	**Adjusted OR** **95%CI**
**Vitamin D concentrations**		
Not deficient (upper three quartiles)	Ref	Ref
Deficient (lowest quartiles)	1.49 (1.08–2.05)	1.53 (1.10–2.12)
**Sex of the baby**		
Boy	Ref	
Girl	0.94 (0.71–1.25)	
**Mother’s age in years**		
< 30	1.17 (0.75–1.83)	
≥ 30	Ref	
**Mother’s education**		
0–5 years	0.92 (0.69–1.22)	
> 5 years	Ref	
Mother’s height		
< 145 cm	1.81 (1.29–2.54)	1.81 (1.27–2.57)
≥ 145 cm	Ref	Ref
**Parity**		
0/Primi	1.53 (1.12–2.10)	1.55 (1.12–2.12)
1 to 3	Ref	Ref
≥ 4	1.33 (0.84–2.09)	1.30 (0.82–2.08)
**Place of delivery**		
Home	1.15 (0.83–1.58)	
Facility	Ref	
**Skilled delivery assistance**		
Skilled birth attendant	Ref	
Untrained	1.18 (0.44–3.14)	
**Taking iron tablets during pregnancy**		
No	Ref	
Yes	1.70 (1.20–2.40)	1.66 (1.17–2.37)
**Tobacco consumption during pregnancy**		
No	Ref	
Yes	1.27 (0.88–1.83)	
**Passive or indirect smoking during pregnancy**		
No	Ref	Ref
Yes	1.47 (1.02–2.13)	1.60 (1.09–2.34)
**Chewing betel leaf during pregnancy**		
No	Ref	
Yes	0.96 (0.72–1.28)	
**Religion**		
Muslim	Ref	
Other's	0.89 (0.58–1.38)	
**Husband’s education**		
0–5 years	1.12 (0.81–1.53)	
> 5 years	Ref	
**Crowding index**		
≤ 2	Ref	
> 2	0.97 (0.71–1.33)	
**Household wealth quintiles**		
Poorest	0.96 (0.67–1.36)	
Middle	1.03 (0.73–1.46)	
Richest	Ref	

## Discussion

In a population-based cohort study of pregnant women followed through the early post-partum period in rural Bangladesh, we documented a high prevalence of serum 25(OH)D deficiency during the second trimester of pregnancy. A vitamin D concentration of < 50 nmol/L or 20 ng/ml is considered deficient and associated with unfavorable outcomes [32]. In the present study, about 3 out of 4 Bangladeshi pregnant women had a vitamin D concentration of < 50 nmol/L. For this analysis, we considered women in the lowest vitamin D quartile with a level of < 30.25 nmol/L as deficient and the remaining women as not deficient although women in the second and third vitamin D quartiles also had a trend towards a higher risk of PTB compared to women in the highest vitamin D quartile. Thus, this analysis showing about a fifty percent higher risk of preterm birth (aOR 1.53 95% CI; 1.10 to 2.12) in VDD women compared to those who were considered not deficient is conservative.

In addition to VDD, maternal short stature, primiparity, passive smoking, and iron supplementation during pregnancy were also significantly associated with higher risks of PTB. The significantly higher risk of PTB among women who consumed iron during pregnancy is counter-intuitive. An earlier study conducted in the same population found a similar result [33]. The benefits of iron supplementation during pregnancy in iron-deficient population are well-established [34]. Results from a systematic review which included 48 randomized trials and 44 cohort studies, revealed a significant effect of prenatal iron consumption on reducing the risk of low birth weight (RR: 0.81; 95% CI: 0.71, 0.93), but the effect on preterm birth was not significant (RR: 0.84; 95% CI: 0.68, 1.03) [34]. There is also evidence to suggest that increasing iron intake is not always beneficial. Iron availability may influence the severity and chronicity of maternal infections and thus might lead to negative pregnancy outcomes, including preterm birth [35], particularly in populations where the prevalence of maternal infections is high.

Our study showed that there was increased risk of PTB among women who had VDD. However, the current literature on vitamin D concentrations and PTB is conflicting. Some studies showed an association while others did not. In a study conducted in South Carolina, USA, using two datasets from National Institute of Child Health and Human Development (*n* = 333) and Thrasher Research Fund (*n* = 154), Wagner et al., 2015 found an increased risk of PTB in women with lower vitamin D concentrations [36]. Women who had vitamin D concentrations < 20 ng/mL in their third trimester had 3.3 times the odds of PTB compared to those who had concentrations > 40 ng/mL [36].

In a retrospective cohort study, also conducted in South Carolina, USA, McDonnell et al., (2017) found that pregnant women who had vitamin D concentrations > 40 ng/mL had 62% lower odds of having PTB when compared to women who had vitamin D concentrations < 20 ng/mL [37]. A Meta-analysis of observational studies conducted in 10,098 women also showed an increased risk of PTB in those who had vitamin D concentrations < 20 ng/mL (OR 1.29, 95% CI: 1.16,1.45) [38].

However, several other studies did not show an association between VDD and PTB. A prospective cohort study by Wang S et al., (2021) in 3,465 pregnant women in Zhoushan Maternal and Child Health Hospital, Zhejiang, China did not show a significant association between maternal VDD and risk for PTB [39]. A retrospective study of 1,1641 women in southern China also did not find an association [40].

Randomized clinical trials (RCT) conducted to assess whether vitamin D supplementation to those who are deficient can improve the rate of PTB has also been inconclusive. Sablok et al., (2015) performed a RCT in 180 pregnant women in Delhi, India and found an 8.3% PTB rate among women who received Vitamin D compared to a rate of 21.1% among those who did not [41]. Rostami et al., (2018) conducted a stratified randomized trial among 2,500 Iranian women and showed a 40% (95% CI, 0.40 to 0.80) lower rate of PTB in women who were supplemented with vitamin D [42]. However, another randomized control trial conducted by Hossain et. al., 2014 at the university hospital in Karachi, Pakistan, did not show an effect. In this study of 207 pregnant women, one group received ferrous sulfate and calcium while the other group received 4,000 IU of Vitamin D3. Though vitamin D concentrations increased in both mother and baby with supplementation, there was no significant difference in PTB rate [43]. Mojibian et al., (2015) conducted a randomized clinical trial in 500 pregnant women (12–16 weeks) with vitamin D deficiency and did not see a significant difference in the PTB rate among those that were given 400 IU of vitamin D daily versus 50,000 IU of vitamin D every 2 weeks [44]. The reason for this conflicting results in different studies are likely multifactorial, including different experimental designs, difference in the timing of measurement of vitamin D level, prevalence of vitamin D deficiency and PTB in the population studied, and different geographical locations and racial backgrounds. The RCTs that showed no benefit in terms of reduction of PTB with supplementation had relatively small sample size. Further RCTs with a larger number of pregnant women in vitamin D deficient population may help delineate whether supplementing mothers can help reduce PTB rate.

The mechanism of maternal vitamin D deficiency and PTB has not been fully elucidated. However, recent studies suggested several pathways including oxidative stress, imbalance in the regulation of the inflammatory response, and compromise of placental function during pregnancy [45, 46]. Yamada et al., (2020) hypothesized that women with VDD may experience increased risk of PTB due to deregulation of immune response [47]. Improved maternal vitamin D status may act through reducing the risk of infections, including bacterial vaginosis that has been implicated in the causation of preterm labor [48].

The strength of the study is its population-based prospective design. The main outcome variable, PTB, was based on gestational age dating by early pregnancy ultrasound conducted by trained ultrasonologists. Thus, the term/preterm classification was accurate compared to classification based on reported date of last menstrual period. The study also has several limitations. This is a case–control study, which can be subject to bias and confounding [49]. Since we collected data prospectively from the entire cohort using the same methods, the bias, if any, should be minimum [50]. Another limitation is that we did not have data on all possible risk factors for PTB.

## Conclusion

The study showed a high prevalence of VDD in Bangladeshi pregnant women, and that these women had an increased risk of PTB. This findings from a case–control study should be cautiously interpreted to infer causality. Additional research is warranted, both observational to further explore the associations between VDD and the risk of PTB and randomized trials to examine if the supplementation is beneficial.

## Data Availability

The dataset used and analyzed for this manuscript will be available from the corresponding author on request.

## Abbreviations

LMICs: Low- and middle-income countries
PTB: Preterm birth
OR: Odds ratio
CI: Confidence interval
AMANHI: Alliance for Maternal and Newborn Health Improvement
CHWs: Community health workers
icddr,b: International center for diarrhoeal disease research Bangladesh
ECLIA: Electrochemiluminescence immunoassay
GA: Gestational age
BMI: Body mass index
AOR: Adjusted odds ratio
NICHD: National Institute of Child Health and Human Development
RCT: Randomized clinical trials
